# Glyoxalase Goes Green: The Expanding Roles of Glyoxalase in Plants

**DOI:** 10.3390/ijms18040898

**Published:** 2017-04-24

**Authors:** Subramanian Sankaranarayanan, Muhammad Jamshed, Abhinandan Kumar, Logan Skori, Sabine Scandola, Tina Wang, David Spiegel, Marcus A. Samuel

**Affiliations:** 1 Department of Biological Sciences, University of Calgary, Calgary AB T2N 1N4, Canada; subbu@itbm.nagoya-u.ac.jp (S.S.); mjamshed@ucalgary.ca (M.J.); abhinandan.kumar2@ucalgary.ca (A.K.); laskori@ucalgary.ca (L.S.); sscandol@ucalgary.ca (S.S.); 2 Institute of Transformative Bio-Molecules (WPI-ITbM), Nagoya University, Furo-cho, Chikusa-ku, Nagoya, Aichi 464-8602, Japan; 3 Department of Chemistry, Yale University, 225 Prospect St., New Haven, CT 06511, USA; tina.wang@yale.edu (T.W.); david.spiegel@yale.edu (D.S.)

**Keywords:** glyoxalase, methylglyoxal, plants, development, stress response

## Abstract

The ubiquitous glyoxalase enzymatic pathway is involved in the detoxification of methylglyoxal (MG), a cytotoxic byproduct of glycolysis. The glyoxalase system has been more extensively studied in animals versus plants. Plant glyoxalases have been primarily associated with stress responses and their overexpression is known to impart tolerance to various abiotic stresses. In plants, glyoxalases exist as multigene families, and new roles for glyoxalases in various developmental and signaling pathways have started to emerge. Glyoxalase-based MG detoxification has now been shown to be important for pollination responses. During self-incompatibility response in Brassicaceae, MG is required to target compatibility factors for proteasomal degradation, while accumulation of glyoxalase leads to MG detoxification and efficient pollination. In this review, we discuss the importance of glyoxalase systems and their emerging biological roles in plants.

## 1. Introduction

All living cells depend on the process of cellular respiration for their energy needs. Despite being the first step in cellular respiration, the process of glycolysis leads to the production of methylglyoxal (MG), a reactive carbonyl (RC) byproduct. The sugar-derived RCs, particularly MG, are cytotoxic: They can react with DNA, proteins, and lipids and modify or disrupt their physiological functions. Cells have evolved the glyoxalase detoxification pathway as a protection against the harmful effects of MG. Glyoxalase I (GLYI) and glyoxalase II (GLYII), are the enzymes of the glyoxalase pathway that catalyze the detoxification of methylglyoxal to non-toxic d-lactate using reduced glutathione as cofactor [1,2]. A shorter route to detoxification of methylglyoxal (in a single step without the requirement of glutathione) mediated by glyoxalase III enzyme has been proposed in a few organisms, but its specific activity has been found to be significantly lower than glyoxalase I [3,4,5].

The glyoxalase system has been well studied in the animal kingdom and numerous roles of glyoxalases, including its function in cell proliferation, embryogenesis, maturation, and cell death have emerged through recent studies [2,6]. GLYI is an important candidate for clinical research, as diseases such as diabetes and hyperglycemia have been associated with increased formation of methylglyoxal [7,8]. Cancerous cells rely on glycolytic metabolism to derive energy for their growth, and GLYI is usually upregulated in these cells. GLYI has been suggested as a useful prognostic factor and a target for the therapy of gastric and pancreatic cancers [9,10].

Interestingly, photosynthetic plants also accumulate MG from sugar metabolism, and this excess MG needs to be eliminated in order to maintain homeostasis [11]. The presence of glyoxalases in the plant kingdom was first reported in Douglas fir needles [12], and since then several studies reported its presence in both monocotyledonous and dicotyledonous plants [13,14,15,16,17,18]. Plant glyoxalases have been primarily associated with various abiotic stress responses and due to their ability to confer tolerance to various stresses, they have been suggested as a biomarker for stress tolerance [18,19]. The existence of glyoxalases as a multigene family in plants suggests the possibility of several undiscovered functional roles for these enzymes. In this article, we discuss the importance of the glyoxalase system in plants and their expanding functional roles with an emphasis on the newly discovered role in the regulation of plant reproduction and protein turnover. We also discuss the roles of methylglyoxal in cellular signaling and present data on imaging of methylglyoxal in plant reproductive tissues, which may become a powerful tool for studies related to MG in plants.

## 2. Methylglyoxal Detoxification System in Plants

**Figure 1:**
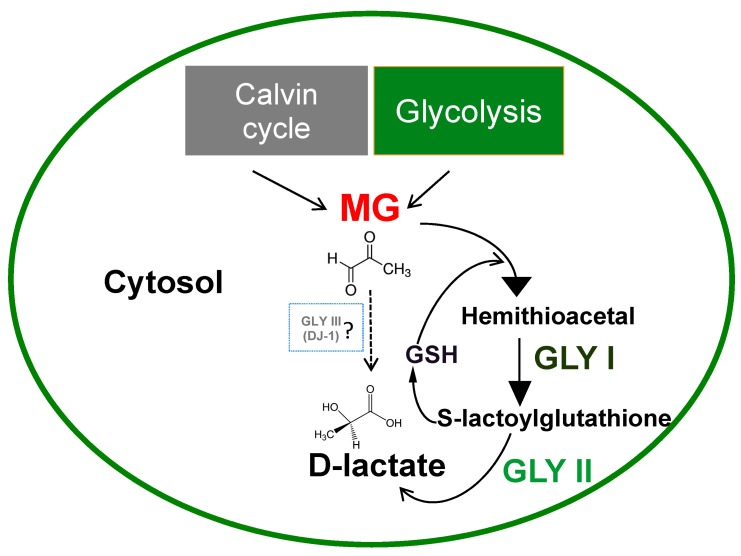
Major routes of methylglyoxal (MG) production and detoxification system in plants. Methylglyoxal is produced as a result of spontaneous dephosphorylation of glyceraldehyde-3 phosphate (G3P) in the Calvin cycle of photosynthesis and from the dephosphorylation of triose phosphate intermediates (TPI) during glycolysis in the cytosol of plant cells. The major routes of enzymatic detoxification of MG in plants are by the two-step glyoxalase pathway mediated by GLYI and GLYII, which depends on the presence of reduced glutathione. A one-step process mediated by GLYIII that does not require reduced glutathione has also been proposed, but the functional role of this enzyme in MG detoxification is debatable.

Plants accumulate high concentrations of sugars in their cells through the process of photosynthesis. Methylglyoxal is thus constantly produced in the plants during glycolysis reactions and in the Calvin cycle of photosynthesis (Figure 1) [11,20]. Under abiotic stress conditions such as salinity, drought, and cold stress, concentrations of MG in plant cells are reported to increase rapidly from 35–75 μM under normal conditions to 200 μM (2- to 6-fold) [21,22]. A consistent increase in MG levels is observed in aging plants. In broccoli (*B. oleracea L* var. *italica*, cv. GDDH33), higher MG levels were observed (3.9 µM) in leaves of 65-day-old plants compared to 9-day-old plants (2.8 µM) [23].

Reliable estimation of MG concentration is challenging and has been found to be highly variable based on the analytical method used. Measurement of methylglyoxal by stable isotopic dilution analysis LC-MS/MS and mathematical metabolic modeling has identified that MG concentration is in the range of 3–4 µM (nmol/g fresh weight) in plant tissues, which is comparable to the concentrations in animal tissues estimated by the same methodology [23]. Therefore, previously reported higher MG levels in plants could possibly be overestimates resulting from inappropriate assay methodology, where triosephosphates degrade to MG during sample processing. Light and dark cycles (diurnal cycles) also contribute to changes in the levels of MG-derived glycation adducts in plants. In Arabidopsis, MG-derived advanced glycation end-product (AGE) residues, N_ε_-caboxyethyl-lysine (CEL), and methylglyoxal–derived lysine dimer (MOLD) have been shown to display an oscillatory, diurnal behavior where maximal residual contents were observed in the middle of light and dark cycles [24]. Given the need to live with consistently high amounts of MG, plants have evolved an efficient system to eliminate this toxic compound and the harmful effects caused by it.

In plants, the MG detoxification process is primarily mediated by the glyoxalase pathway comprising of enzymes glyoxalase I (GLYI/GLO1, S-D lactoylglutathione lyase) and glyoxalase II (GLYII; hydroxyacylglutathione hydrolase) that sequentially convert MG to d-lactate in a two-step process (Figure 1). Glyoxalase I acts by isomerizing hemithioacetal formed by the spontaneous combination of MG and GSH leading to the formation of S-lactoylglutathione, which is then hydrolyzed to d-lactate by glyoxalase II, regenerating GSH in the process [1,25]. The first enzyme of this pathway, GLYI is usually a metalloenzyme that requires metal ions for its activation. All of the prokaryotic GLYI enzymes were believed to be Ni^2+^-dependent, while eukaryotic GLYI were believed to be Zn^2+^-dependent. However, recent studies have pointed to the existence of Ni^2+^ and Zn^2+^-dependent GLYI candidates in both prokaryotes and eukaryotes [26]. A rice glyoxalase I enzyme involved in detoxification of methylglyoxal in the nucleus has been discovered recently that does not show any strict metal ion dependence for its activity [27]. The second enzyme of the pathway, GLYII also requires divalent cations for its activity and has a binuclear metal binding center that incorporates iron and zinc [28,29].

Unlike most microbial and animal systems, in plants, glyoxalases exist as a multigene family [19,30]. It has been reported that rice has a total of 19 GLYI proteins encoded by 11 genes and 4 GLYII proteins encoded by three genes [26,30]; while the model plant Arabidopsis has 22 GLYI proteins encoded by 11 genes and 9 GLYII proteins encoded by five genes [30]. A genome study carried out in *Glycine max* (soybean) also identified 24 *GLYI* and 12 *GLYII* genes [31]. The molecular mechanism, subcellular localization, and functional roles of these diverse isoforms are yet to be uncovered. The existence of multiple forms of these genes in plants probably indicates the possible diverse, tissue-specific roles glyoxalases may have, besides detoxification of MG.

Recent discoveries have proposed a single step detoxification of methylglyoxal to d-lactate by a unique glyoxalase pathway consisting of glyoxalase III enzyme that does not require any cofactor (Figure 1). This class of enzyme was first identified in *E. coli* and was reported to be a member of DJ-1/Pfp1 superfamily with a conserved catalytic triad His-Cys-Glu in its active site [3,32]. Recent research has identified the existence of DJ-1/Pfp1 domain containing proteins in the plant kingdom and proposed the presence of glyoxalase III, as a shorter route to MG detoxification [4,33]. Expression profiling of *OsDJ-1* genes in rice under various abiotic stresses revealed that these genes are upregulated by a multitude of stresses including dicarbonyl stress (exogenous MG) [4]. Furthermore, the enzyme activity of one of the highly-expressed members, *OsDJ-1C*, showed that it could utilize MG as substrate to produce d-lactate in a glutathione-independent manner. Site-directed mutagenesis of the conserved cysteine in the N-terminal domain of this protein resulted in the loss of GLYIII activity, reconfirming it to be a functional enzyme [4]. Contrary to the conventional GLYI/II enzymes, GLYIII enzymes required higher substrate concentrations and had very low specific activity, suggesting that GLYI proteins are the primary enzymes to detoxify MG under normal physiological conditions and GLYIII comes into action under extreme stress conditions where plants accumulate massive amounts of MG [4].

Recently, concerns were raised over the involvement of DJ-1 (GLYIII) protein in MG detoxification. Human DJ-1 proteins have 10,000-fold reduced specific activity compared to human GLO1, suggesting that DJ-1 is unlikely to contribute to the cellular MG detoxification system in mammals [5]. Further, DJ-1 protein in Drosophila did not display any glyoxalase activity and it has been reported that the previously identified deglycation activity of DJ-1 is likely an artifact produced by the use of Tris buffer in the assay system [34]. Therefore, any assumptions based on involvement of DJ-1 in MG detoxification mechanisms should be made with an element of caution.

Non-glyoxalase enzymes can also contribute another route to detoxification of methylglyoxal. Aldose/aldehyde reductase (ALR) and aldo-keto reductase (AKR) can reduce methylglyoxal to acetol. Over-expressing *ALR* or *AKR* genes in plants can confer tolerance to various abiotic and heavy metal stresses [35,36,37]. Rice plants over-expressing AKR displayed increased tolerance to oxidative and heat stress by accumulating lower levels of methylglyoxal [37]. In addition to these enzymes, aldehyde dehydrogenase (ADH) has been shown to detoxify MG by catalyzing its conversion into pyruvate, which can then enter the tricarboxylic acid cycle (TCA) [38].

## 3. Functional Roles of Glyoxalases in Plants: From the Past to the Present

In the last three decades, numerous studies have reported various functional roles for glyoxalases, including its well-established roles in conferring abiotic stress tolerance. The existence of glyoxalases as a multigene family in plants suggests that there could be several more undiscovered roles and tissue-specific functions for glyoxalases. Recent research by our group has uncovered a role for glyoxalase I in regulating pollen–pistil interactions. In this section, the different functional roles that have been assigned to plant glyoxalases are discussed (see also Table 1).

### 3.1. Glyoxalase as a Marker for Cell Division

The earliest evidence of glyoxalase and its relation to cell division comes from studies carried out with germinating pea, where its activity was found to increase with an increase in indole acetic acid levels (auxin) and was found to be inhibited by colchicine [39]. Increase in glyoxalase I activity was also correlated with an increase in cell growth, protein, and DNA synthesis in *Datura* callus, while addition of mitotic inhibitors to the growth medium resulted in inhibition of glyoxalase I activity [40]. Proliferative cell suspension cultures of soybean (*Glycine max* L.) exhibited increased glyoxalase activity during the logarithmic growth phase, which decreased with inhibition of cell division, making glyoxalase a molecular marker for cell division [16]. Even though there are reports that link glyoxalase and cell division in plants, the causal relationship between the increase in glyoxalase activity and cell division still remains unknown.

### 3.2. Glyoxalase as a Mitigator of Abiotic Stress

Due to their sessile nature, plants constantly combat different environmental stresses such as drought, extreme temperatures, salinity, and heavy metal toxicity. Plants have evolved several innate mechanisms that enable them to thrive under these extreme conditions. The glyoxalase system is one amongst these mechanisms that allows plants to cope with increasing MG levels, which accumulates to toxic concentrations when exposed to various abiotic stresses [21].

*GLYI* transcript levels show a 2- to 3-fold increase in the roots, stems, and leaves of tomato plants treated with NaCl, mannitol, or ABA [41]. Similarly, *GLYI* expression was significantly upregulated on exposure to salinity, mannitol, and heavy metal stress in *Brassica juncea* [42]. In pumpkin seedlings, *GLYI* was upregulated on exposure to white light, salinity, MG, and heavy metal stress [43]; in wheat, upon exposure to ZnCl_2_ [44], and in rice, following exposure to arsenite [45]. Like *GLYI*, expression of *GLYII* also increases with exposure to different stresses such as salinity, heavy metals, and ABA [46]. In rice, exposure to treatments such as salinity, desiccation, extreme temperatures, ABA, and salicylic acid led to an increase in *GLYII* expression [47]. *GLYII* transcript levels also increased upon exposure to ZnCl_2_ in *Brassica juncea* [46]. Treatment of roots of Arabidopsis seedlings, with the xenobiotic compound, 2,4,6-trinitrotoluene (TNT) also resulted in an increase in *GLYII* transcripts [48]. Genome-wide expression studies in Arabidopsis and rice have revealed a differential response of multi-gene family of glyoxalases to various stresses in different tissues and during different growth and reproductive stages [30].

In silico analysis of Arabidopsis *GLYII* has revealed that it is co-expressed with stress responsive genes, and several cis-regulatory elements associated with stress inducible genes were found upstream of *GLYII* [49]. Similarly, genome-wide analysis and expression profiling studies of glyoxalase gene families in soybean (*Glycine max*) have revealed their likely involvement in regulation of abiotic stress responses [31]. Several stress-responsive cis elements such as abscisic acid responsive element (ABRE), auxin responsive element (AuxxRR-core), ethylene responsive elements, and heat shock element (HSE) have been identified in the promoter regions of both *GmGLYI* and *GmGLYII* family members, suggesting that these genes could be regulated by hormonal and stress response pathways [31].

Transgenic approaches in various plant models have demonstrated the ability of glyoxalases in imparting abiotic stress tolerance. In one of the earliest studies, the transformation of tobacco with a *GLYI* gene from *Brassica juncea* resulted in transgenic tobacco plants with tolerance to high levels of NaCl and MG [42]. Overexpression of a glyoxalase gene, *OsGly I*, conferred abiotic stress tolerance to NaCl, ZnCl_2_, and mannitol, and improved grain yield in rice [50]. Similarly, overexpression of *GLYII* in tobacco, rice, and mustard also rendered the plants tolerant to high levels of MG and NaCl [51,52,53,54]. Double transgenic lines of tobacco, overexpressing both *GLYI* and *GLYII*, were found to be more tolerant to salinity than plants overexpressing either *GLYI* or *GLYII* [51]. The same approach was also used in tomato to enhance resistance to salinity stress [55]. The glyoxalase pathway has also been engineered in plants for heavy metal tolerance. Transgenic tobacco plants expressing the glyoxalase pathway were tolerant to a toxic concentration of ZnCl_2_ and other heavy metals such as cadmium and lead [56]. From these studies, it is clear that overexpression of either the entire glyoxalase pathway or a specific glyoxalase enzyme has the potential to confer tolerance to a wide range of abiotic stresses.

### 3.3. Glyoxalases and Biotic Stress

In addition to abiotic stresses, plants also encounter biotic stresses and damage from living organisms such as bacteria, fungi, viruses, parasites, and insects. Glyoxalase pathway genes are quite sensitive to biotic stresses. *GLYI* was found to be downregulated upon infection of rice with *Xanthomonas oryzae* (causative organism for rice blight disease) or *Pyricularia grisea* (rice blast fungus) [57]. Contrary to this, *GLYI* was rapidly induced in response to *Fusarium graminearum* infection in wheat, which reached its peak (2.3-fold) 12 h after inoculation, suggesting a role of *GLYI* during Fusarium head blight (FHB) infection [44]. A similar increase of *GLYI* transcript (3-fold) and protein was observed when *Brassica napus* was infected with the fungal pathogen *Sclerotinia sclerotiorum* [58]. Transgenic rice plants overexpressing cecropin A, a gene that confers resistance to rice blast fungus showed an ~6-fold induction in *GLYI* levels compared to wild-type rice [59]. The promoter regions of *GmGLYI* and *GmGLYII* genes also have several biotic stress-responsive cis elements such as the fungal elicitor responsive element (BOX-W1), jasmonate elicitor responsive element (JERE), defense and stress responsive element (TC-rich), wounding and pathogen responsive elements (W-box and WUN-motif), salicylic acid responsive element (TCA) and methyl jasmonate-responsive elements (CGTCA box and TGACG motif) [31]. Further studies have to be undertaken to determine if overexpression of glyoxalase genes could offer direct protection to plants from pathogens.

### 3.4. Glyoxalase as a Regulator of Plant Reproduction

Many plant species possess the ability to recognize and reject its own pollen to avoid genetic defects and inbreeding depression in the offspring. This ability is brought about by a molecular mechanism called self-incompatibility (SI).

**Figure 2:**
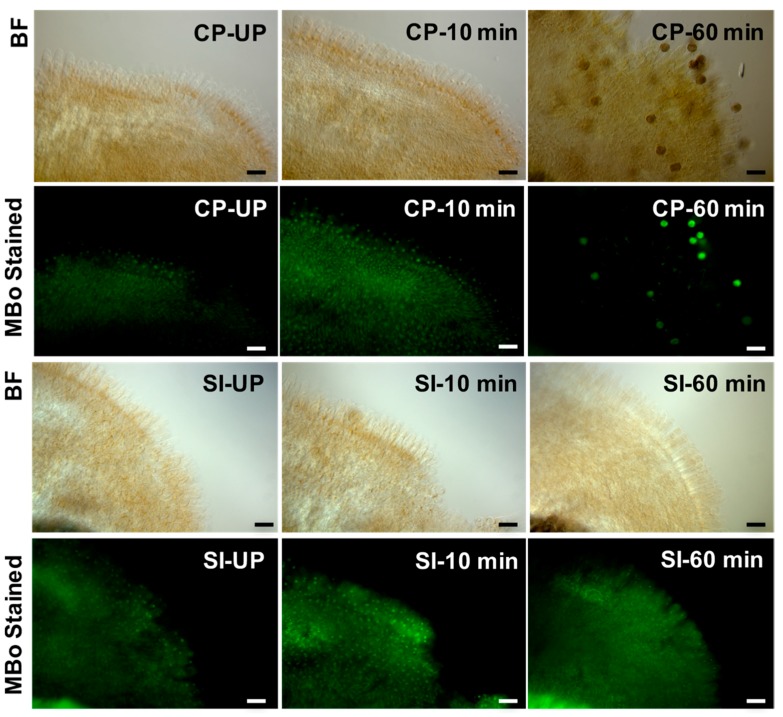
Visualization of MBo-stained papillary cells after 0, 10, and 60 min of pollination, suggesting an increase in MG levels after 10 min in both Westar (compatibly pollinated) and W1 (self-incompatible pollination), which decreases after 60 min of compatible pollination, but persists following self-incompatible pollination. (BF: bright field; CP: compatible pollination; SI: self-incompatible pollination; UP: unpollinated), scale bars = 50 µm.

SI in *Brassica* (sporophytic self-incompatibility) results from an allele-specific interaction between the pollen encoded small cysteine-rich, secreted protein (SCR/SP11) and the stigmatic S-receptor kinase (SRK), which leads to the activation of Armadillo Repeat-Containing 1 (ARC1) E3 ubiquitin ligase, which targets compatibility factors needed for the successful pollination for proteasomal degradation [61,62,63,64,65]. In contrast, following compatible pollination (CP), the resources or compatibility factors are provided by the stigma to the dry pollen so that pollen tubes can germinate and penetrate the stigmatic cuticle for successful fertilization [66,67]. Using a DIGE (difference gel electrophoresis)-based proteomics assay, we had previously identified glyoxalase I (GLO1) as one of the major candidates downregulated following SI response in *Brassica napus* [68,69]. Functional analysis of the role of GLO1 during pollination using isogenic *Brassica napus* lines (self-compatible Westar and the self-incompatible W1) revealed that GLO1 activity is essential for compatible pollination to occur [60]. Biochemical assays to measure GLO1 activity and levels revealed a significant increase in GLO1 activity and levels following compatible pollinations (CP) within 60 min and a significant reduction in GLO1 activity and levels within 60 min after SI pollinations. When MG levels were determined in the stigmas using either a spectrophotometric approach or fluorescent imaging using a fluorescent sensor MBo (methyl diaminobenzene-BODIPY) [70] (see supplemental material), MG levels correlated with the GLO1 protein/activity following CP and SI. MG accumulation could be observed in W1 stigmas as early as 30–60 min of incompatible pollination, while after compatible pollination, MG levels rapidly increased around 10 min and returned to unpollinated levels by 60 min (Figure 2). The increase in MG levels in stigmas following landing of the pollen is probably the result of increased glycolysis to generate energy for supporting the growing pollen tube. The growth of the pollen tube through the female tissue can be compared to the growth of fungal hyphae in plant cells and in both cases, plant cells would experience some stress leading to MG accumulation. Although MBo was previously studied in detail both in vitro and in eukaryotic cells [66], this is the first application of MBo in plants. Therefore, fluorescence due to interactions with non-MGO metabolites cannot be precluded, and all interpretations arising from MBo studies must be asserted with this caveat in mind.

**Figure 3:**
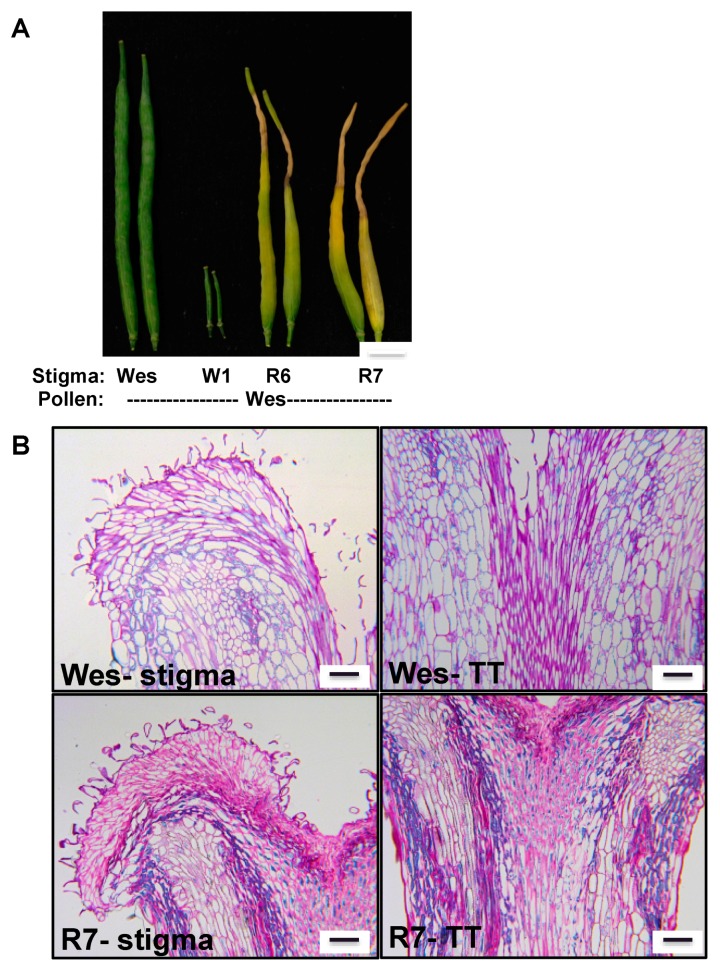
Loss of *GLO1* results in increased damaged to the stylar transmitting tract and female reproductive tissue during pod development. (**A**) *GLO1* suppressed lines (R6 and R7) showing tissue death phenotype that radiates down from the top of the stigma during pod development following compatible pollination; (**B**) Histological sections of pods of similar age from Wes and R7 line showing increased cell death in the vascular tissue and cells around the stigma and stylar transmitting tract (TT) in the absence of *GLO1.* Scale bars = 100 µm.

Stigma-specific RNAi suppression of *GLO1* in compatible *B. napus* Westar lines resulted in a significant reduction in pollen attachment and seed set, indicating the role of GLO1 as a compatibility factor to mediate successful pollination [60]. Interestingly, in the strong RNAi lines, during pod growth, we consistently observed development of a zone of tissue death, on the top half of the pod that radiated from the stigma (Figure 3A). Sectioning of these pods revealed that the cells of the vasculature and transmitting tract had collapsed in the *GLO1*-suppressed lines when compared to control Westar lines (Figure 3B) (see supplemental material). Although a speculation at this point, we believe that, following compatible pollination, the rapid increase in MG in the stigma should be regulated and detoxified to prevent it from leaking and migrating through the vasculature into the reproductive structures such as the funiculus and the ovules. If uncontrolled and not restricted at the stigma, as observed in the RNAi lines, excess MG can migrate into the reproductive tract, leading to localized cell death in these zones during early pod development. Such zones will manifest as a region of dead tissue in mature pods. Based on these observations, we propose that, although MG functions cell-autonomously in regulating pollination in the papillary cells of the stigma, a non-cell-autonomous function in the transmitting tract is also completely possible.

Converse to the RNAi lines, overexpression of GLO1 in self-incompatible W1 lines resulted in a partial breakdown of SI response by bringing down MG levels in the stigmas [60]. Further experiments revealed that GLO1 is targeted for proteasomal degradation by ARC1 during self-incompatible response, which leads to rapid accumulation of MG and MG-modified proteins in the stigma [60].

**Figure 4:**
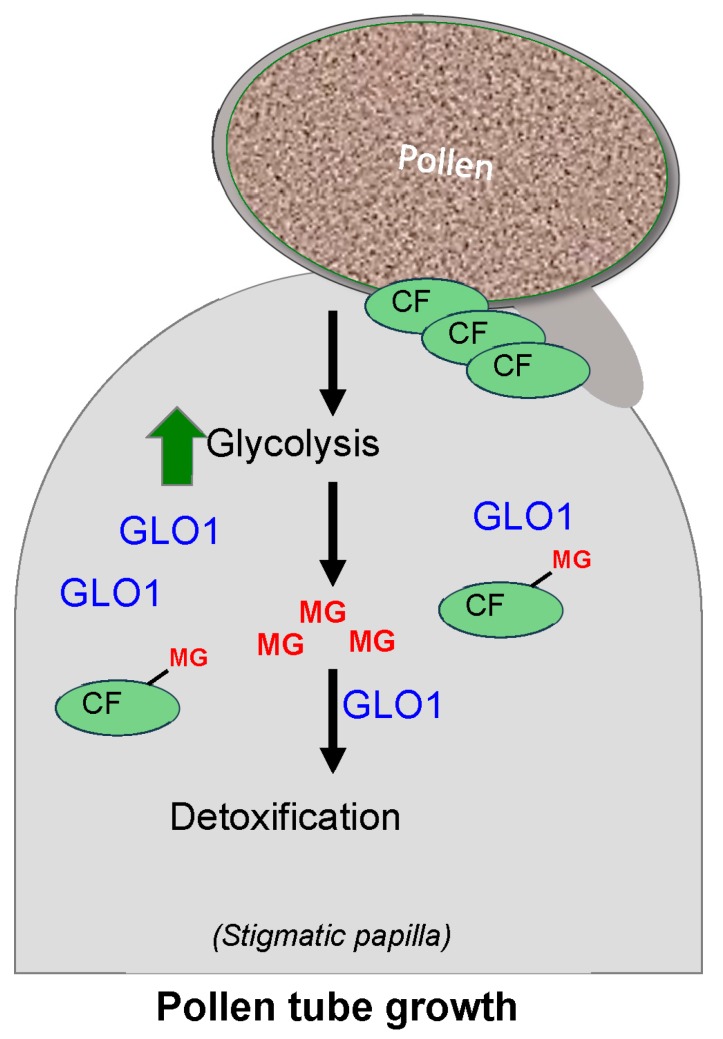
Detoxification of methylglyoxal (MG) by GLO1 ensures successful pollination. Landing of pollen on stigmatic papillae leads to increased glycolysis resulting in accumulation of toxic MG; MG can modify compatibility factors (CFs) to trigger their degradation. GLO1 protein levels are increased following compatible pollination, which results in rapid detoxification of MG, allowing CFs to be delivered to the site of pollen attachment, ensuring successful pollination.

Depletion of essential factors required for pollination occurs during SI as a result of rapid degradation of MG-modified compatibility factors by hyper-activated ARC1, resulting in pollen rejection response [60]. In case of compatible pollination, immediately after pollination, the rate of glycolysis goes up to provide energy for the growing pollen and leads to rapid accumulation of MG in the papillary cells. This rapid increase in MG causes a transient abrogation of proteasome function due to sudden increase in MG levels. This, combined with the lack of hyper-activation of ARC1 in the absence of SI response, results in the stabilization of the compatibility factors and facilitation of the delivery of these factors at the pollen attachment site [60]. In order to mitigate the increase in MG levels following compatible pollination, GLO1 protein levels also go up with time in the papillary cells receiving compatible pollen, which counteracts the effect of MG, resulting in successful pollination (Figure 4; [60]).

**Figure 5:**
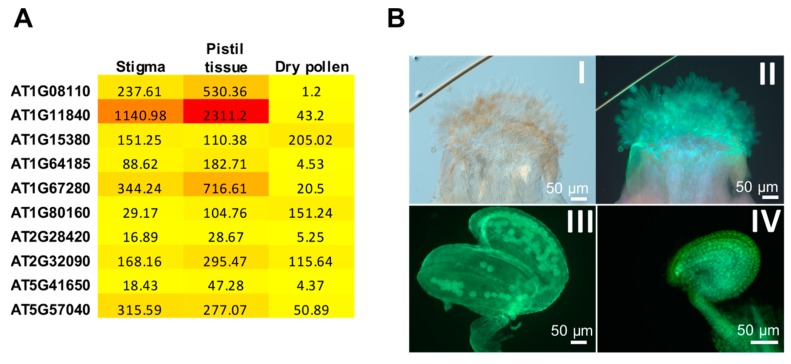

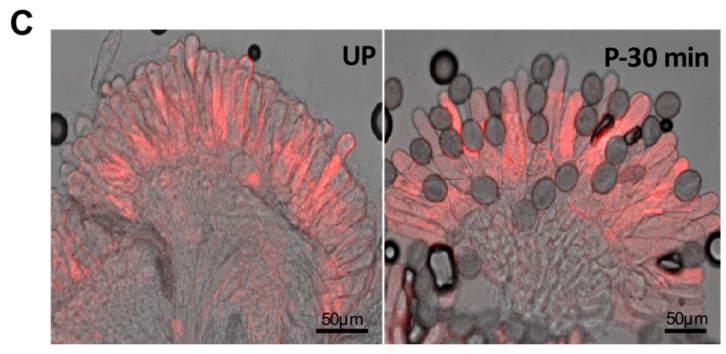
Expression analysis of glyoxalase I family members and imaging of methylglyoxal in reproductive tissues of Arabidopsis. (**A**) Heat maps representing the absolute expression values of glyoxalase genes in Arabidopsis stigma, pistil tissue (consisting of ovary) and dry pollen from ATH1 genome array. Values were generated using BAR Expression Browser (**B**) Visualization of methylglyoxal through MBo staining: (I) DIC image of an Arabidopsis stigma; (II) Stigma stained with MBo; (III) Anther and pollen stained with MBo; (IV) Female gametophyte stained with MBo; (**C**) Comparison of intensity of BnGLO1-RFP in Arabidopsis stigmas before and after 30 min of pollination through confocal microscopy. Scale bars = 50 µm.

In Arabidopsis, expression analysis of glyoxalase I family members in reproductive tissues (stigma, pistil, and pollen) revealed a highly differential expression (Figure 5A). Methylglyoxal staining of Arabidopsis reproductive tissues with MBo revealed high levels of MG in pistils, anther, pollen, and the female gametophyte, suggesting the existence of differentially expressed glyoxalase family members in these tissues for MG detoxification (Figure 5B). Furthermore, pollination enhances the expression of *Bn*GLO1-RFP (*Brassica napus Glyoxalase I*) in Arabidopsis stigmas (Figure 5C), reaffirming the essential role of glyoxalase in detoxifying MG during pollination.

### 3.5. Roles of MG in Cellular Signaling

Several studies have explored the role of methylglyoxal (MG) in cellular signaling in plants, and it has been proposed that MG might be acting as a signaling molecule in plants [19,71]. MG could function like a plant hormone and replace kinetin to initiate differentiation of plantlets from calli in *Solanum nigrum* and *Daucus carota* [72]. MG has a positive effect on differentiation and shoot morphogenesis of tobacco callus (*Nicotiana tabacum* L.) [73]. MG is also an inhibitor of seed germination and root elongation in plants [74]. MG is known to induce expression of stress-responsive transcription factors *RD29B* and *RAB18* in a concentration-dependent manner through an ABA-dependent pathway [74]. In many of these experiments with exogenous application of MG, the concentration of MG used (0.1–10 mM) is 25–2500-fold higher than the physiological levels found in plants, raising the possibility that the observed changes are likely the result of acute intoxication of plant tissues by exogenous MG. We also have to take into consideration that, during exogenous application of chemicals to plant tissues, it is typical to apply higher concentrations in order to observe a physiological effect. This is due to the fact that efficiency of uptake of the chemicals into the target cell past the various layers of the plant tissue is usually poor and the physiological effects are mostly subtle.

MG has also been linked to opening and closing of stomata in plants. MG induces stomatal closure by inducing ROS accumulation in the guard cells via the Ca^2+^-dependent pathway and inhibits light-induced stomatal opening by inhibiting K^+^ influx into guard cells, which happens by a modification of the C-terminal region of the KAT1 potassium channel [75]. Data from our studies on pod development in *glyoxalase I* suppressed transgenic Brassica lines suggest that MG could work in a non-cell-autonomous manner to bring about developmental changes. This hypothesis needs further experimental verification.

MG-mediated protein modifications can have tremendous implications in cellular signaling. For example, we have shown that MG-modified proteins are more prone to proteasomal degradation [60], which could affect turnover of major proteins that may be involved in different signaling cascades, causing disruption of normal cellular function and development.

## 4. Conclusions

Several new functional roles of glyoxalases in plants have emerged in recent years. Early research on soybean glyoxalases revealed its involvement in the process of cell growth and organ differentiation [16]. Transcriptomic and proteomic studies coupled with transgenic approaches have enabled the establishment of a role for glyoxalases in conferring abiotic stress tolerance. Though there is evidence that glyoxalases function in biotic stress responses, it remains to be demonstrated that they have a role in defense against plant pathogens.

The recent discovery of the role of *glyoxalase I* in regulating pollen–pistil interactions is just a new beginning in understanding the functions of these enzymes in plant reproduction. Given that MG is highly abundant in plant reproductive tissues, especially in the female gametophyte as revealed by MBo staining (Figure 5B), it would not be surprising if glyoxalases played a role in the final step of pollen tube reception or egg and sperm cell fusion.

Since MG has various functional roles in plants, it is doubtless that glyoxalases, through their ability to detoxify MG, would also have related functional roles in plant growth and development. With the rapid advancements in genomics, proteomics, metabolomics, live cell imaging, and genome editing technologies, new and exciting roles for MG and glyoxalases in plants are bound to emerge. Nevertheless, studies on MG and glyoxalases to date have expanded our understanding of the glyoxalase system in plants and have provided the tool set to further explore the roles of this intriguing toxic metabolite and its detoxification counterpart, the glyoxalase pathway.

## Figures and Tables

**Table 1:** Biological roles of glyoxalases in plants

Biological role	Plant species	References
Marker for cell division	Pea (*Pisum sativum*), Datura, Soybean (*Glycine max* L.)	[16,39,40]
Mitigator of abiotic stress (Salinity, Drought, Mannitol, ABA, extreme temperatures and heavy metal stress)	Tomato (*Solanum lycopersicum*), Indian Mustard (*Brassica juncea*), Rice (Oryza sativa), Soybean (*Glycine max*), Tobacco (Nicotiana tabacum) , Pumpkin and *Arabidopsis*	[30,31,41,42,43,44,45,46,47,48,49,50,51,52,53,54,55,56,57]
Biotic stress tolerance (Evidence obtained mainly from expression analysis of fungal infected plants and promoter analysis of the genes of Glyoxalase family)	Rice (*Oryza sativa*), Wheat (*Triticum aestivum*), Canola (*Brassica napus*), Soybean (*Glycine max*)	[31,57,58,59]
Involvement in Plant reproduction	Canola (*Brassica napus*), *Arabidopsis*	[60], this paper
Regulation of protein turn-over (Indirectly by regulating MG levels)	Canola (*Brassica napus*)	[60]

## Supplementary Materials

Supplementary materials can be found at www.mdpi.com/1422-0067/18/4/898/s1.
