# Supplementary material for: Glyoxalase Goes Green: The Expanding Roles of Glyoxalase in Plants
Source: Int J Mol Sci. 2017 Apr 24;18(4):898. doi: 10.3390/ijms18040898 (PMC5412477; doi:10.3390/ijms18040898)
Supplement: Supplementary file 1 [file ijms-18-00898-s001.pdf]

Supplemental Data

Materials and Methods

MBo Staining of Stigmas and Reproductive tissues:

Freshly opened flowers of Westar (compatible) and W1 (self-incompatible) canola lines were harvested at 0, 10 and 60 minutes after pollination and fixed in 500 µL of 3 (ethanol): 1 (acetic acid) for 30 minutes. After three washes with water, the stigmas were softened in 500 µL of 1 N NaOH at 65 ^{0}C followed by three washes with water. The stigmas were then incubated in 20 µM of MBo stain for 30 minutes in the dark, followed by visualization under green channel (GFP setting) using the Leica epifluorescence microscope.
For the MBo staining of Arabidopsis reproductive tissues, stage 12 flowers were collected from *Arabidopsis thaliana* (*Col-0*) and fixed as described previously followed from treatment with 1N NaOH at 50 ^{0}C for 1 hour and three subsequent washes with water. Anthers, stigmas and ovules (female gametophyte) were separated out from the flowers using a fine forceps and incubated in 20 µM of MBo stain for 30 minutes in dark followed by visualization under an epifluorescence microscope. Fixed but unstained tissues were used as controls for this experiment.

Histological Analysis of Pods from *GLO1* RNAi lines:

Developing pods from *GLO1* suppressed RNAi lines (R6 and R7) and Westar control lines were collected and fixed for 48 hours in 1.6 % paraformaldehyde, 2.5 % glutaraldehyde in phosphate buffer (pH 6.9). Following fixation tissue samples were dehydrated for embedding using an alcohol series. Samples were infiltrated with Technovit® 7100 and blocked for sectioning. Sections were cut to 3 µm using a Reichert-Jung 2040 Autocut rotary microtome with glass Ralph knives. The thin sections were stained with Toluidine Blue-O and photographed. Further details for histological preparations can be found in Yeung et al., (2015).

Yeung, E.C.T., Stasolla, C., Sumner, M.J., and Huang, B.Q. (2015). Plant microtechniques and protocols (Springer: Cham).

Confocal Microscopy of BnGLO1-RFP in Arabidopsis stigmas:

Arabidopsis stigmas expressing BnGLO1-RFP were imaged using the Leica SP5 confocal microscope. Stage 12, unpollinated and stage 13 pollinated stigmas were mounted in 50% glycerol and images were obtained with 40X oil immersion objective lens. To acquire RFP signals stigmas were scanned using HeNe 543 LASER (excitation 543; emission 585-649). RFP and DIC images were overlaid to produce the merged image.
